# NanoCon: contrastive learning-based deep hybrid network for nanopore methylation detection

**DOI:** 10.1093/bioinformatics/btae046

**Published:** 2024-02-01

**Authors:** Chenglin Yin, Ruheng Wang, Jianbo Qiao, Hua Shi, Hongliang Duan, Xinbo Jiang, Saisai Teng, Leyi Wei

**Affiliations:** School of Software, Shandong University, Jinan, China; Joint SDU-NTU Centre for Artificial Intelligence Research (C-FAIR), Shandong University, Jinan, China; School of Software, Shandong University, Jinan, China; Joint SDU-NTU Centre for Artificial Intelligence Research (C-FAIR), Shandong University, Jinan, China; School of Software, Shandong University, Jinan, China; Joint SDU-NTU Centre for Artificial Intelligence Research (C-FAIR), Shandong University, Jinan, China; School of Opto-electronic and Communication Engineering, Xiamen University of Technology, Xiamen, China; Faculty of Applied Sciences, Macao Polytechnic University, Macao 999078, China; School of Qilu Transportation, Shandong University, Jinan, China; School of Software, Shandong University, Jinan, China; Joint SDU-NTU Centre for Artificial Intelligence Research (C-FAIR), Shandong University, Jinan, China; School of Software, Shandong University, Jinan, China

## Abstract

**Motivation:**

5-Methylcytosine (5mC), a fundamental element of DNA methylation in eukaryotes, plays a vital role in gene expression regulation, embryonic development, and other biological processes. Although several computational methods have been proposed for detecting the base modifications in DNA like 5mC sites from Nanopore sequencing data, they face challenges including sensitivity to noise, and ignoring the imbalanced distribution of methylation sites in real-world scenarios.

**Results:**

Here, we develop NanoCon, a deep hybrid network coupled with contrastive learning strategy to detect 5mC methylation sites from Nanopore reads. In particular, we adopted a contrastive learning module to alleviate the issues caused by imbalanced data distribution in nanopore sequencing, offering a more accurate and robust detection of 5mC sites. Evaluation results demonstrate that NanoCon outperforms existing methods, highlighting its potential as a valuable tool in genomic sequencing and methylation prediction. In addition, we also verified the effectiveness of our representation learning ability on two datasets by visualizing the dimension reduction of the features of methylation and nonmethylation sites from our NanoCon. Furthermore, cross-species and cross-5mC methylation motifs experiments indicated the robustness and the ability to perform transfer learning of our model. We hope this work can contribute to the community by providing a powerful and reliable solution for 5mC site detection in genomic studies.

**Availability and implementation:**

The project code is available at https://github.com/Challis-yin/NanoCon.

## 1 Introduction

5-Methylcytosine (5mC) is the predominant form of DNA methylation found in eukaryotes, playing a crucial role in biological processes such as gene expression regulation, embryonic development, and X chromosome inactivation(Moore *et al.* 2013, Schübeler 2015, Zhang *et al.* 2018, Li *et al.* 2022). Traditional sequencing methods, such as bisulfite sequencing, involve chemical treatments where DNA samples are exposed to a bisulfite solution; this solution reacts with unmethylated cytosines (C), converting them to uracil (U), while methylated forms, such as 5mC and 5-hydroxymethylcytosine (5hmC), remain unchanged during the process, allowing for subsequent comparison and identification of methylation sites(Frommer *et al.* 1992, Neri *et al.* 2016). While bisulfite sequencing is indeed capable of accurately identifying the specific locations of methylation, this method may potentially damage DNA, impacting the quality and accuracy of sequencing data. In addition, in certain cases, it may not distinguish between different types of methylation modifications, such as 5mC and 5hmC.

Nanopore sequencing is an emerging genomic sequencing technology that detects changes in electrical currents generated by DNA or RNA molecules passing through nanopores to read molecular sequences(Deamer *et al.* 2016). Compared to traditional methods, nanopore sequencing has the ability to directly detect DNA methylation without the need for chemical sample treatment. During nanopore sequencing, methylated and unmethylated cytosines induce distinct electrical current signals as they pass through the nanopore (Wang *et al.* 2014, Liu *et al.* 2021, Tourancheau *et al.* 2021). By comparing these signals, we can directly detect the methylation status of DNA. Nanopore sequencing offers advantages such as unlimited read length, real-time sequencing, amplification-free analysis, and direct detection of modifications(Simpson *et al.* 2017, Xu and Seki 2020, Wang *et al.* 2021).

Methods used for 5mC site detection in nanopore sequencing data can be roughly categorized into three main groups: (i) direct statistical analysis of electrical signals, (ii) traditional machine learning methods, and (iii) deep learning methods. For example, Tombo (Stoiber *et al.* 2016), a statistical analysis method, conduct the identification of potential modification sites by analyzing irregularities in the electrical signal. The advantage of statistical analysis methods lies in their simple and straightforward processes, making it suitable for preliminary detection of methylation sites. However, these methods can be sensitive to noise and significant signal fluctuations in the data, which can lead to a certain degree of false positives and false negatives. As a traditional machine learning tool, Nanopolish (Simpson *et al.* 2017) utilized a Hidden Markov Model to detect methylation signals and statistical models to assess the methylation status of each locus.

Traditional machine learning methods often rely on manually selected features, referred to as “hand-crafted features.” These features, while informed by domain knowledge, may limit performance in handling complex, high-dimensional data scenarios. Thus, for large-scale datasets, the analysis time of these methods can be considerably prolonged. In recent years, deep learning methods have made significant advancements in methylation site prediction, not only for 5mC sites but also for other biological sequence sites such as A-I (Nguyen *et al.* 2022) or 6 mA (Liu *et al.* 2019, Qin *et al.* 2022) sites, demonstrating excellent performance in handling complex methylation patterns and across different datasets (Simpson *et al.* 2017, Bonet *et al.* 2022, Cheetham *et al.* 2022). For example, DeepSignal-plant (Ni *et al.* 2021), a deep learning tool, can detect genome-wide 5mCs of three sequence contexts (i.e. CpG, CHG, and CHH) in plants from Nanopore reads. Moreover, methBERT (Zhang *et al.* 2021), a nonrecurrent modeling approach, explored the Bidirectional Encoder Representations from Transformers (BERT) model for nanopore methylation detection. Beyond these, METEORE (Yuen *et al.* 2021) (MEthylation deTEction with nanopORE sequencing) stands out as a comprehensive tool that integrates multiple deep learning methods for Nanopore methylation detection, providing an aggregated prediction result. Furthermore, Remora (Nanopore 2022), featuring a convolutional neural network (CNN) architecture, is crucial for detecting DNA modifications like 5mC through Oxford Nanopore Technologies (ONT) sequencing. The Remora models from ONT are now widely adopted for methylation calling and are accessible via basecallers like Guppy, dorado, and bonito. Though previously available through Megalodon, this pathway is deprecated, highlighting the dynamic evolution in DNA modification detection technology.

In real-world scenarios, the distribution of methylated and nonmethylated sites in processed nanopore sequencing datasets is imbalanced, sometimes severely. Existing methods have used various strategies to improve model training performance, including oversampling methods that increase the number of methylated sites through operations like shifting, and undersampling methods that reduce the number of nonmethylated sites through negative sampling, aiming to construct a balanced dataset. However, these methods do not fully capture the real-world data distribution. In particular, undersampling may result in the loss of important samples, leading to the increase of model bias, while oversampling increases the number of similar samples, raising the risk of overfitting (Thabtah *et al.* 2020).

To address these challenges, in this study, we developed a contrastive learning-based deep hybrid neural network called NanoCon for the detection of 5mC sites in nanopore sequencing data. Evaluation on the benchmark datasets shows that our proposed model outperforms state-of-the-art methods for 5mC methylation prediction. In particular, we incorporated the Transformer (Vaswani *et al.* 2017) model with the 5-mer representation strategy to encode the sequence information and a fully connected neural network to encode the electrical signal data, and integrated them using the (Bidirectional Gated Recurrent Unit) Bi-GRU (Dey and Salem 2017) model. To tackle the imbalanced dataset problem in nanopore sequencing methylation prediction, we used the contrastive learning strategy to capture more discriminative feature representations between the methylation sites and nonmethylation sites, demonstrating that our model has great potential to be a powerful and practically useful deep learning tool for 5mC methylation prediction. Moreover, cross-species and cross-5mC methylation motifs experiments demonstrated the robustness and stability of our model. In addition, the visualization of feature distributions among different methods intuitively indicates that our model can learn high-quality representations of methylation sites compared with other deep-learning methods.

## 2 Materials and methods

### 2.1 Datasets

In this study, we followed the same processing methodology as described in the DeepSignal-plant for the aforementioned procedures. Specifically, we utilized the datasets of *Arabidopsis thaliana* (*A.thaliana*) and *Oryza sativa* (*O.sativa*), as curated by Peng Ni *et al.* We obtained the genome reference for *A.thaliana* from NCBI, specifically using version GCF_000001735.4_TAIR10.1 (Wheeler *et al.* 2007). As for *O.sativa*, we downloaded the genome reference and gene annotation from EnsemblPlants, utilizing version IRGSP-1.0 (Howe *et al.* 2020). Building upon these datasets, we added new human data to our study. DNA nanopore reads for the individual *NA12878* were obtained from the dataset processed by Jain *et al.* (2018). These nanopore reads can be accessed and downloaded from the European Nucleotide Archive (ENA) under the accession number PRJEB23027. In addition, bisulfite sequencing analysis results for Homo sapiens *NA12878* were obtained from the ENCODE project. The specific dataset used in this study is available under accession number ENCFF835NTC (Consortium 2012). This inclusion of human data provides a comparative angle to our research, potentially elucidating unique or conserved patterns across species. These datasets encompassed nanopore sequencing results and bisulfite sequencing results. Randomly selecting 20 000 reads for each species, we constructed the datasets and used bisulfite sequencing as the gold standard for labeling motifs.

**Figure 1.**
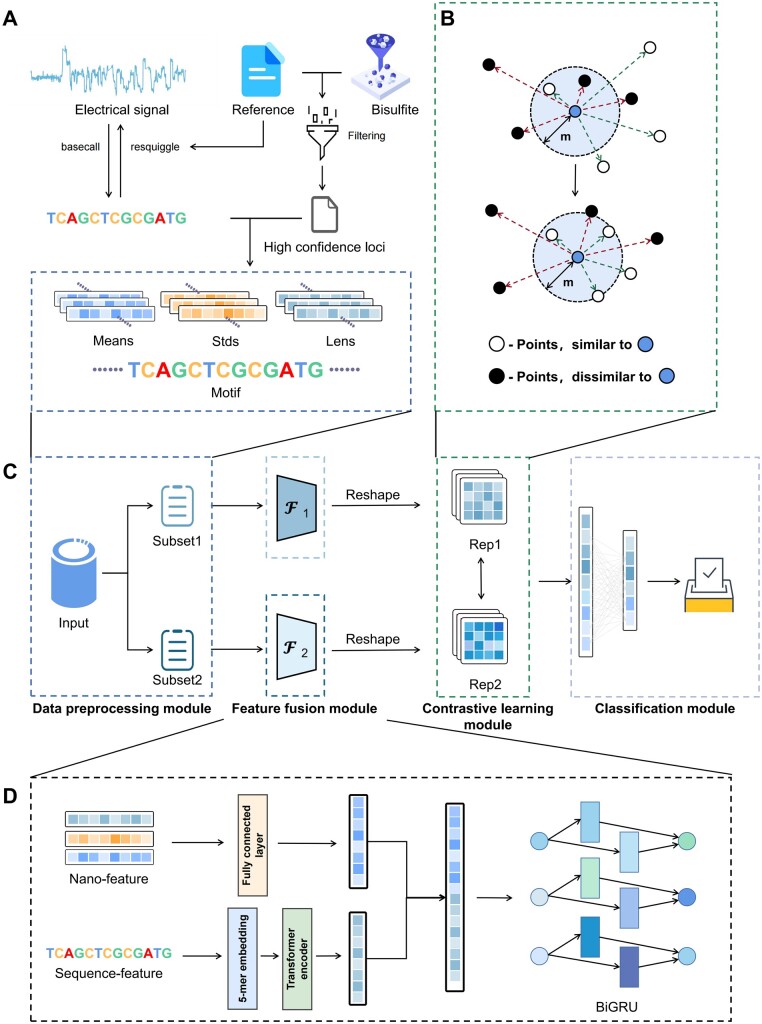
The overall architecture of NanoCon. (A) The process of constructing datasets from sequencing data. (B) The visualization of contrastive learning. Through contrastive learning, we can draw the sample points with same class closer and distance the points that are not of the same class. (C) The overall process of our deep learning model. Firstly, we divided the data into two parts, which are separately input into identical feature fusion module to obtain the corresponding feature representations. These two representations are then used for contrastive learning and input into the classification module to obtain the final prediction. (D) The specific construction of the feature fusion module. By fusing the DNA sequence features extracted from Transformer model and nanopore electrical signal features from fully connected neural network using Bi-GRU model, we obtain deeper and more robust feature representations.

Figure 1A details our data processing workflow. Initially, basecalling is performed on nanopore sequencing data using Guppy, followed by alignment to a reference genome with the Tombo tool. For bisulfite sequencing data, alignment to a reference is also conducted, and high-quality methylation sites are identified using Bismark. Positive sites are defined by a coverage above 5 and a methylation rate over 90%, while negative sites have a 0% methylation rate. Adopting this strategy enables us to extract a maximal number of high-credibility positive examples and even more credible negative examples. Subsequently, we extract positive and negative instances from nanopore sequencing for the training set. The data instances include five elements: site positional information in the genome, motif base sequence from basecalling, mean electrical signal of the motif, standard deviation of this signal, and the signal's length. We define the length of the motif as 13, with methylated and unmethylated bases located at the central positions of the motif. The detailed statistics of the resulting datasets are summarized in Table 1. It can be observed that the distribution of positive and negative examples within the dataset varies across different topics and species. Moreover, in the case of *A.thaliana* and *O.sativa*, the negative examples significantly outnumber the positive ones, whereas in the *NA12878* dataset, the quantity of positive examples exceeds that of the negative examples, albeit with a more balanced ratio between the two.

**Table 1.** The number of methylated and nonmethylated sites in the dataset.

Species	Methylated sites	Unmethylated sites	Proportion
*A.thaliana*	208 177	10 227 828	0.02035398
*O.sativa*	1 796 190	6 263 834	0.28675568
*NA12878*	2 509 760	1 356 171	1.85062208

### 2.2 The architecture of our model

The NanoCon model, as shown in Fig. 1, is a deep learning-based model that leverages a hybrid structure of the Transformer and Bi-GRU for nanopore methylation detection. It utilizes contrastive loss and cross-entropy loss during training process, which can be divided into four modules: (i) data preprocessing module, (ii) feature fusion module, (iii) contrastive learning module, and (iv) classification module. In the data preprocessing module, we divide the raw biological data into two subsets for contrastive learning tasks. Each subset contains sequence data and corresponding auxiliary data (nanopore electrical signal data). We use a custom data collation function to organize and assemble the data into batches, facilitating model training. The feature fusion module is the core component of the model, responsible for integrating the sequence data and auxiliary data to extract more meaningful feature representations. We utilize a series of neural network models for feature fusion, including Transformer model and Bi-GRU model. The Transformer encoder layers encode and model the sequence data, while the Bi-GRU layers handle the combined representation of sequence data and auxiliary data. The contrastive learning module is primarily concerned with the calculation of the contrastive loss. Two separate data inputs are processed through the feature fusion module to generate two sets of representations. These representations are then passed to the contrastive loss function which calculates the loss based on the cosine similarity between the representations. The classification module consists of several linear layers that transform the feature representations into a form suitable for classification. The transformed representations are then passed to a cross-entropy loss function which calculates the classification loss.

### 2.3 Data preprocessing module

As shown in Fig. 1C, prior to data input, we randomly split the input data into two equal parts. This process ensures that both parts have an equal number of samples. By dividing the data into two parts, we create pairs of samples where each pair consists of one sample from each split. These pairs serve as input for the contrastive learning framework, allowing us to compare the similarity or dissimilarity between the representations generated by the model. This enables us to explore the relationship between different samples and learn meaningful representations that capture the underlying structure of the data.

### 2.4 Feature fusion module

The feature fusion module is a core component of our proposed NanoCon, responsible for extracting informative representations from the input genetic sequence and nanopore electrical signal data, and further merging these features. At the beginning of the process, the input genetic sequence data are processed through a tokenizer with the 5-mer representation strategy (Ji *et al.* 2021). We selected this particular 5-mer tokenizer due to its unique suitability for our data, a choice informed by the fact that nanopores used in sequencing can accommodate five base pairs. The tokenizer adeptly converts the raw genetic sequences into a form that is well-suited for processing by Transformer encoder layers.

Next, the Transformer encoder processes the tokenized sequences, generating comprehensive, high-dimensional representations of the input genetic sequences. This is achieved through the utilization of a powerful mechanism known as the self-attention mechanism, or the transformer attention mechanism. The self-attention mechanism enables the encoder to capture the interdependencies between different positions within the input sequences, allowing the model to focus on relevant parts while considering the context of the entire sequence. It assigns different weights or attention scores to each token based on its relevance to other tokens. The attention mechanism can be mathematically represented as follows:

        Q=XWQK=XWKV=XWV           (1)

        SelfAttentionQ, K, V=softmaxQKTdk V   (2)

Here, X represents the embedding of input sequences, and Q, K, V are the query matrix, key matrix, and value matrix, respectively. They are obtained from X through linear transformations using three weight matrices $W^{Q}$, $W^{K}$, and $W^{V}$.

Concurrently, the input nanopore electrical signal data are fed into a Fully Connected (FC) network. This network comprises multiple layers of neurons, with each layer performing a linear transformation and a subsequent nonlinear activation function. The FC network is responsible for encoding the nanopore signal data into a set of informative features.

Following the extraction of features from both the genetic sequence and nanopore signal data, the subsequent step is feature fusion. The features obtained from both sources are concatenated together, forming a comprehensive representation encapsulating information from both the genetic sequence and nanopore data. After concatenation, the combined features are passed through a Bidirectional Gated Recurrent Unit (Bi-GRU). The choice of Bi-GRU stems from its proficiency in handling sequential data and its capacity to capture both past (backward direction) and future (forward direction) dependencies in the context of input sequences. The Bi-GRU processes the concatenated features, thereby integrating the information from both sources into a single, unified representation that will be used for classification in the subsequent module.

To summarize, the feature fusion module leverages the strengths of various neural network architectures, including Transformer encoders, fully connected neural networks, and Bi-GRUs, to extract and integrate information from diverse data types. This architecture ensures that the resulting representations are both informative and comprehensive, thereby enhancing the model's subsequent classification performance.

### 2.5 Contrastive learning module

The main objective of the contrastive learning module is to learn representations of sample features that place similar samples close in the feature space while placing dissimilar ones far apart. This objective is achieved through a specialized loss function known as the Contrastive Loss function. This function propels model learning by capitalizing on the disparities between the sample feature representations.

In the context of contrastive learning, the key idea of the Contrastive Loss function is to compel the model to position samples of the same class in close proximity within the feature space, while samples from different classes should be dispersed into more distant regions. The mathematical expression of this function is as follows:

(3) Losscontrastive=12N∑1-y×Dxi, xj2      +y×Maxmargin-Dxi,xj, 02

In the equation above: N denotes the batch size, $x_{i},\;x_{j}\;$represent the two samples in a pair for contrastive learning, $D\left(x_{i},\;x_{j}\right)\;$symbolizes the distance between the feature representations of $x_{i},\;x_{j}$, y is a binary label where y = 1 when $x_{i},\;x_{j}$ originate from the same class, and y = 0 otherwise, and margin is a predefined distance threshold which we set to 2.

From this, it is clear that for samples from the same class, we desire their feature representation distance D to be as small as possible. For samples from different classes, we expect their feature representation distance to be at least greater than the margin. This encapsulates the essence of the Contrastive Loss function. The distance D in our framework is measured using cosine similarity, which is formulated as follows:

(4) Dxi, xj=xi·xjxi×xj

In this equation, $x_{i},\;x_{j}$ are two vectors, where $\left|x_{i}\right|$ and $|x_{j}|\;$represent the Euclidean length (or 2-norm) of vectors $x_{i}$ and $x_{j}$ respectively, and $x_{i}\cdot x_{j}$ denotes the dot product of vectors $x_{i}\;$and $x_{j}$. By using cosine similarity, our model focuses more on the relative directional relationship between features, thereby mitigating the impact of feature length. This approach effectively captures and compares inherent data patterns, proving instrumental in our contrastive learning scheme.

### 2.6 Classification module

The prediction and classification module act as the final phase of our learning scheme, leveraging the feature representations learned from the contrastive learning module to predict the class of input samples. In this module, we use a Cross-Entropy Loss function to optimize the classification task. The Cross-Entropy Loss function essentially measures the dissimilarity between the model's predicted class probabilities and the true class labels. The mathematical representation of the Cross-Entropy Loss function is as follows:

(5) LossCE=1N∑i=1N-yi·log⁡pi+1-yi·log⁡1-pi

where $y_{i}\;$denotes the true class labels, $p_{i}$ represents the predicted class probabilities from the model, and N is the number of samples.

In our combined learning framework, the total loss used to optimize the model is a weighted sum of the Contrastive Loss from the contrastive learning module and the Cross-Entropy Loss from the classification module. The equation for this is as follows:

(6) Lossfinally=α×Losscontrastive+1-α×LossCE

where ${\mathrm{Loss}}_{\mathrm{contrastive}}$ denotes the Contrastive Loss, ${\mathrm{Loss}}_{\mathrm{CE}}$ signifies the Cross-Entropy Loss, and α is weighting factors that balance the contribution of the two losses which we set it to 0.8.

Importantly, to focus the learning on the task at hand, we adopt a “freezing” strategy during training. In the contrastive learning phase, we freeze the parameters of the classification module, and vice versa. By doing so, we can ensure that the optimization of each module does not interfere with the other, providing a more targeted and efficient learning process. This meticulous orchestration of contrastive learning and prediction classification, supplemented by the freezing strategy, endows our model with the ability to effectively learn from and adapt to complex data patterns.

### 2.7 Performance metrics

Due to the imbalanced nature of our dataset, where there is a significant disparity in the number of positive and negative samples, relying solely on accuracy as an evaluation metric may lead to biases. In this experiment, we have selected five evaluation metrics to comprehensively assess the model's performance: accuracy (ACC), Precision, Recall, F1 score, area under the precision–recall curve (AUPRC), and area under the receiver operating characteristic curve (AUROC). These metrics can be calculated using the following formulas:

(7) ACC=TP+TNTP+TN+FP+FNPrecision=TPTP+FPRecall=TPTP+FNF1Score=2×Precision×RecallPrecision+Recall

By incorporating these diverse evaluation metrics, we can comprehensively assess the model's performance on the imbalanced dataset. The selection of these metrics is based on a holistic consideration of the classification task, ensuring a comprehensive and objective understanding of the model's performance.

### 2.8 Experimental settings

We performed model training and evaluation using the PyTorch Lightning framework on a server equipped with four NVIDIA GeForce RTX 3090 graphics cards. We utilized the Adam optimizer to optimize the model parameters and used a learning rate decay strategy to control the learning process. The model was trained for 100 epochs, and based on our observations, the model achieved its best performance on the validation set around 30 epochs. To alleviate overfitting, we introduced a Dropout layer in the GRU model. These settings were carefully chosen to ensure the accuracy, reliability, and reproducibility of the experiment, thereby enabling a reliable assessment of the model's performance.

## 3 Results

### 3.1 Comparison results on benchmark datasets

In this study, we conducted an evaluation of our proposed deep learning model using three distinct datasets: two from the plant species *A.thaliana* and *O.sativa*, and one from a human dataset. For detailed information on the sources and characteristics of these datasets, please refer to the “Materials and methods” section, under the heading “Dataset.” Our primary comparison was against two established genomics models: The Recurrent Neural Network (RNN) and methBERT. In addition, we compared these models with a CNN-based architecture, similar to Remora, which is recognized for its application in detecting DNA modifications through Oxford Nanopore Technologies. Notably, while Remora's trained CNN models are based on CG (CpG) motifs, necessitating retraining on our dataset.

**Figure 2.**
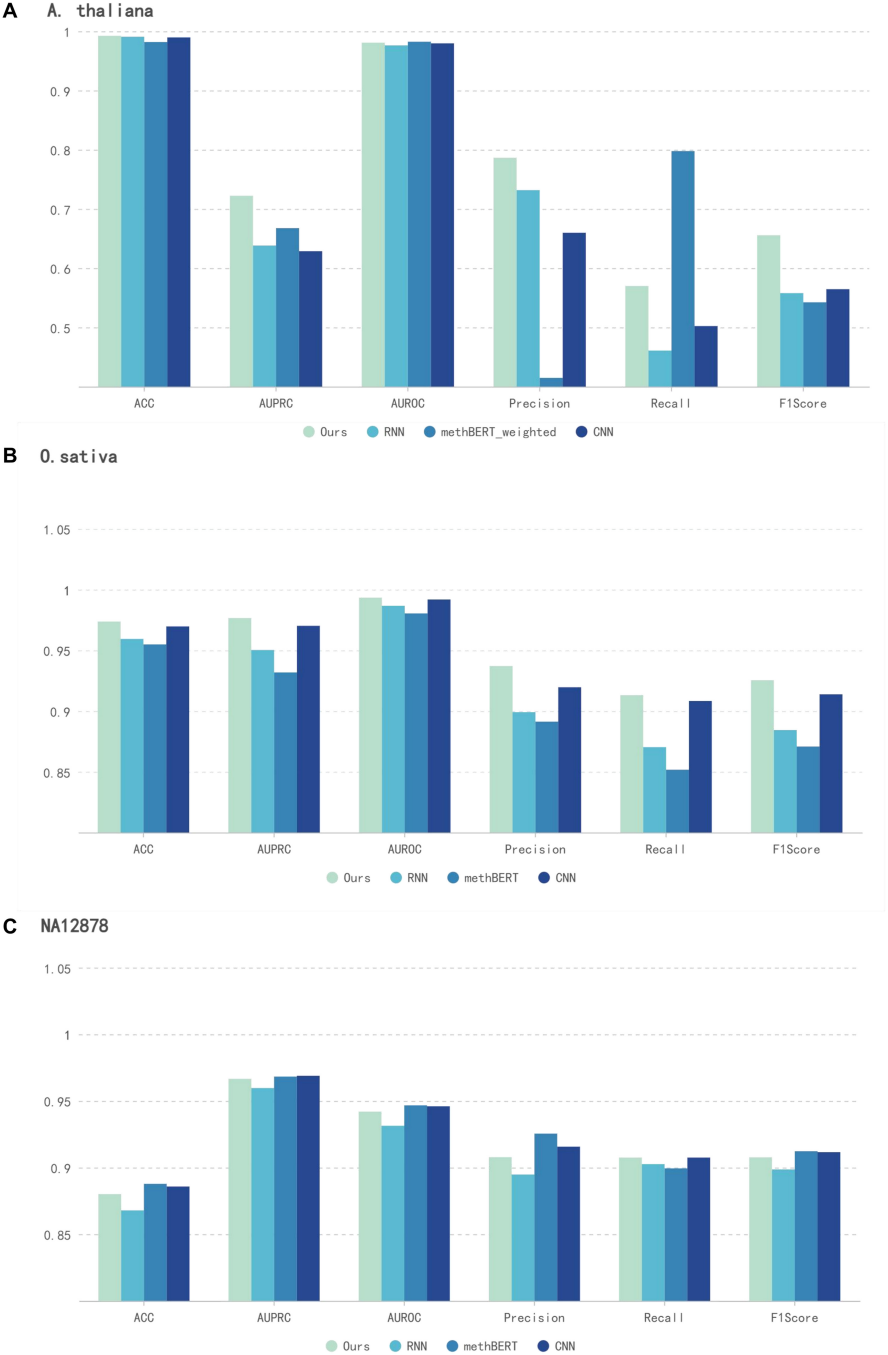
Comparison of the performance of our NanoCon and other methods. (A) Performance comparison of the three models on the *A.thaliana* dataset. (B) Performance comparison of the three models on the *O.sativa* dataset.

To ensure fair evaluation, each dataset was strictly divided into training, validation, and testing sets in an 8:1:1 ratio. This partitioning scheme allows us to leverage the overall nature of the data while avoiding overfitting, with the aim of obtaining more reliable experimental results during the testing phase. Next, we will provide a detailed introduction to the performance of these models on different datasets and explore various factors that may influence their performance. The objective of this study is to identify which model performs better in specific tasks through such comparative experiments, thereby providing valuable guidance and insights for further machine learning research. As demonstrated in Fig. 2 and Table 2, NanoCon outperforms other models in most metrics on both datasets, indicating its superior discriminative ability in dealing with imbalanced datasets.

**Table 2.** Performance of the four models on the three datasets.

	Models	ACC	AUPRC	AUROC	Precision	Recall	F1Score
*A.thaliana*	Ours	0.9924	0.7223	0.9809	0.7865	0.5699	0.6557
	RNN	0.9909	0.6383	0.9763	0.7318	0.4608	0.5579
	methBERT_weighted	0.9819	0.6676	0.9824	0.4147	0.7978	0.5424
	CNN	0.9897	0.6286	0.9797	0.6597	0.5022	0.5645
*O.sativa*	Ours	0.9737	0.9766	0.9934	0.9371	0.9131	0.9254
	RNN	0.9594	0.9503	0.9867	0.8991	0.8703	0.8844
	methBERT	0.9549	0.9318	0.9804	0.8913	0.8517	0.8708
	CNN	0.9697	0.9702	0.9919	0.9196	0.9083	0.9138
*NA12878*	Ours	0.8801	0.9666	0.9420	0.9078	0.9076	0.9077
	RNN	0.8679	0.9597	0.9314	0.8948	0.9026	0.8986
	methBERT	0.8878	0.9683	0.9467	0.9255	0.8994	0.9123
	CNN	0.8858	0.9689	0.9460	0.9157	0.9075	0.9116

On the *A.thaliana* dataset (Fig. 2A), our model achieves the best results in ACC, AUPRC, Precision, and F1 Score, outperforming other models by 0.15%, 5.47%, 5.46%, and 9.16% respectively. However, it was surpassed by the methBERT-weighted model in terms of AUROC and Recall, with a very close AUROC between them. This is because during the training process, we found that the original methBERT model obtained poor performance on the imbalanced *A.thaliana* dataset due to its inability to capture useful information of the real distribution. Consequently, we modified the original methBERT by giving the minority samples higher weight according to the data distribution to make the model pay more attention to them. This also made the methBERT-weigthed model more sensitive to positive instances, resulting in more negative instances being classified as positive, leading to a higher Recall but lower Precision. RNN and CNN models demonstrate performance that is closely matched, however, they still fall short of the efficacy achieved by our model.

On the *O.sativa* dataset (Fig. 2B), our model achieves better results in all the metrics, outperforming other models by 0.4% in ACC, 0.62% in AUPRC, 0.15% in AUROC, 1.75% in Precision, 0.48% in Recall, and 1.16% in F1 Score respectively. Among these, the CNN model's performance metrics were the closest to our model, while it still maintained a substantial lead over both the RNN and methBERT models. It is worth noting that all models perform better on this dataset compared to the *A.thaliana* dataset. This may be due to the fact that the *O.sativa* dataset is more balanced, and the features of the dataset are more easily captured by the models.

In the analysis of the *NA12878* dataset (Fig. 2C), our model showed notable achievements, recording 0.8801 in ACC, 0.9666 in AUPRC, 0.9420 in AUROC, 0.9078 in Precision, 0.9076 in Recall, and 0.9077 in F1 score. While our model outperformed the RNN model in all metrics, with improvements of 1.22% in ACC and 0.69% in AUPRC, it demonstrated mixed results when compared to methBERT and CNN. Specifically, methBERT exhibited a marginal lead in AUPRC and AUROC, likely reflecting its enhanced data distribution sensitivity. It also showed a 1.77% higher precision, indicating more accurate positive sample prediction, though our model achieved slightly better recall. The CNN model, meanwhile, edged out our model in AUPRC and AUROC, albeit with slightly lower precision, recall, and F1 score, reflecting its stable classification capabilities but slightly less precise identification of positive samples. This comparative analysis underscores the strengths and areas for improvement of our model, offering valuable insights for future enhancements.

In summary, our analysis highlights NanoCon’s exceptional performance in managing imbalanced datasets, showcasing its superior classification abilities in these challenging conditions.

### 3.2 Ablation study of NanoCon

**Figure 3.**
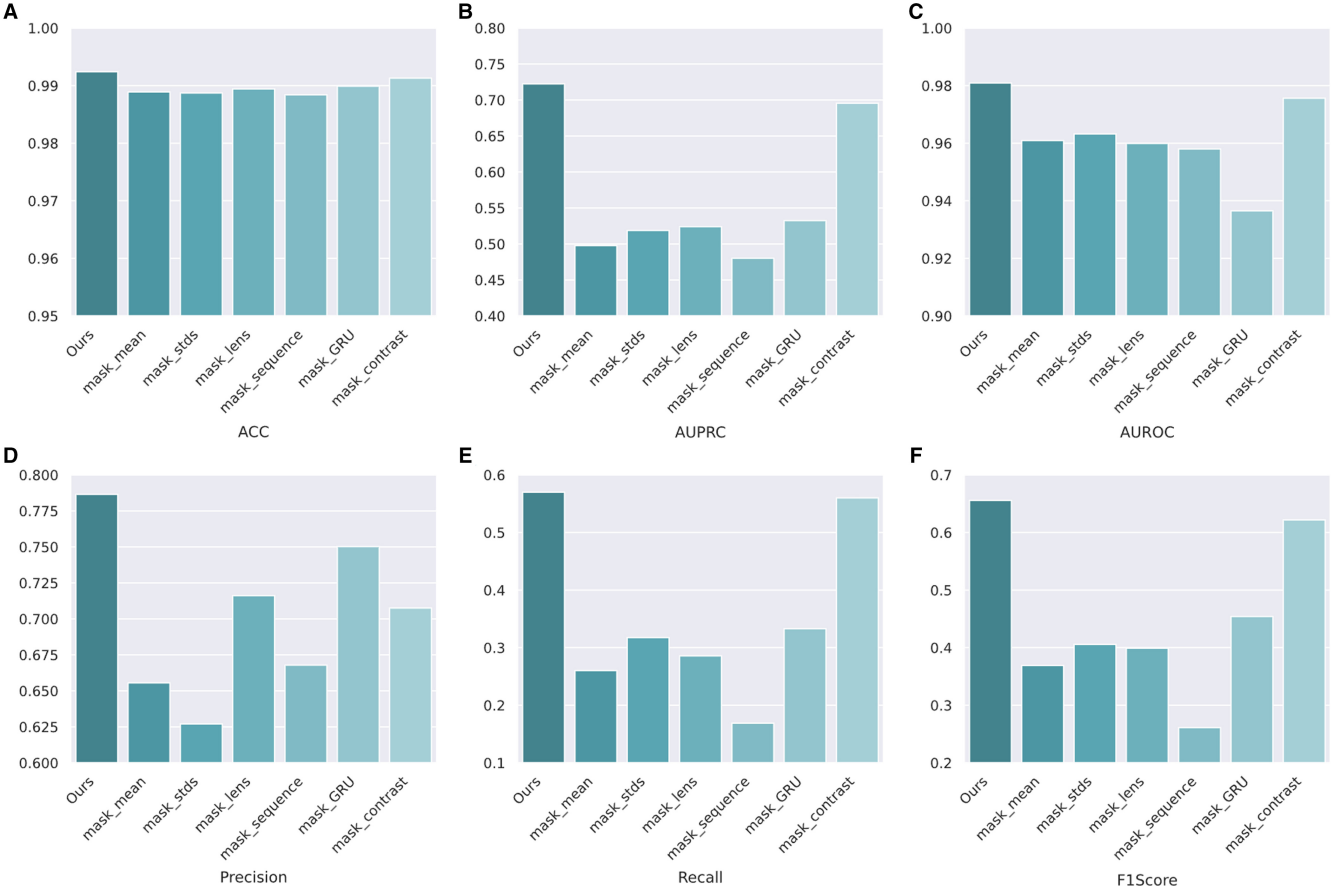
Ablation study of our NanoCon. (A–F) The performance comparison of our NanoCon and its variants on the metrics of ACC, AUPRC, AUROC, Precision, Recall, and F1 Score.

In this section, we explored how changes of input data and network architecture impact the performance of our model. In our dataset, each sample of input data is composed of four parts: a DNA sequence, the corresponding mean electric signal of the base, the standard deviation, and length. By selectively masking these parts, preventing their participation in the model's training, we assess the influence of different sections of the dataset on the model's learning process. For the ablation of model architecture, we curated two model variants: one involves replacing the final Bi-GRU layer with a fully connected layer in the feature fusion module, and the other involves completely removing the contrastive learning module. The aim of these experiments is to gain deeper insights into how modifications to the model's structure affect its overall performance. As shown in Fig. 3A–F and Table 3, we demonstrate the impact on model performance when different sections of the *A.thaliana* dataset are masked and various elements of the model are modified. Given the imbalanced nature of the dataset, we are particularly interested in changes to the AUPRC, Precision, Recall, and F1 Score metrics.

**Table 3.** Performance of our model under different parts of the input data masked and the model component masked.

*A.thaliana*	ACC	AUPRC	AUROC	Precision	Recall	F1Score
Ours	0.9924	0.7223	0.9809	0.7865	0.5699	0.6557
mask_means	0.9889	0.4977	0.9609	0.6555	0.2602	0.3690
mask_stds	0.9887	0.5188	0.9632	0.6270	0.3175	0.4056
mask_lens	0.9894	0.5240	0.9599	0.7161	0.2855	0.3990
mask_sequence	0.9884	0.4802	0.9580	0.6678	0.1689	0.2611
mask_GRU	0.9899	0.5324	0.9365	0.7502	0.3327	0.4540
mask_contrast	0.9913	0.6954	0.9756	0.7075	0.5600	0.6217

As expected, the model that accepted no modifications achieved the best performance across all metrics, indicating that the parts mentioned above each contribute to the final performance of the model. Upon examining the results of masking different parts of the dataset, we can see that the most significant performance drop occurs when the mean electric signal and DNA sequence sections are masked. Meanwhile, masking the length of the electric signal results in the smallest decrease in the AUPRC metric. This could be due to the inherent value the electric signal mean and the DNA sequence data provide to the learning process. These data types contain intricate patterns and comprehensive information about the genetic sequences, contributing significantly to the model's ability to learn and make accurate predictions.

In the experiments comparing different model architectures, the model exhibited a more significant performance decline after replacing the Bi-GRU layer. This suggests that the Bi-GRU layer plays a vital role in feature fusion and extraction within the model, making it more challenging to discern between positive and negative examples when this layer is altered. Without the Bi-GRU layer, the model may struggle to capture the differences and complex dependencies within the genetic sequence data. When the contrastive learning module is removed, there is a significant drop in the Precision metric. This implies that the contrastive learning module greatly contributes to the model's ability to accurately classify sequences by enhancing the discriminative power of the feature representations.

In summary, our experimental results reveal that each component of the dataset and the architecture plays a critical role in the overall model performance. Any modifications, whether masking parts of the dataset or adjusting the model architecture, can lead to a significant shift in performance. This underscores the importance of careful design and thorough testing in bioinformatic model construction.

### 3.3 Evaluating the robustness of NanoCon

In our research, we focused on examining the transfer learning ability of our model across different species and among various DNA methylation motifs within a species. We chose *A.thaliana* and *O.sativa* for cross-species analysis, merging their validation and test sets for evaluation. Our study also investigated the identification of DNA methylation motifs, particularly 5mC in CG (CpG), CHG, and CHH contexts (‘H’ denotes A, C, or T), using the *O.sativa* dataset divided into these categories. Experiments were conducted to analyze cross-methylation motifs in a manner akin to our cross-species experiments. The aim was to deepen our understanding of the model's transfer learning capabilities in a rigorous context.

**Figure 4.**
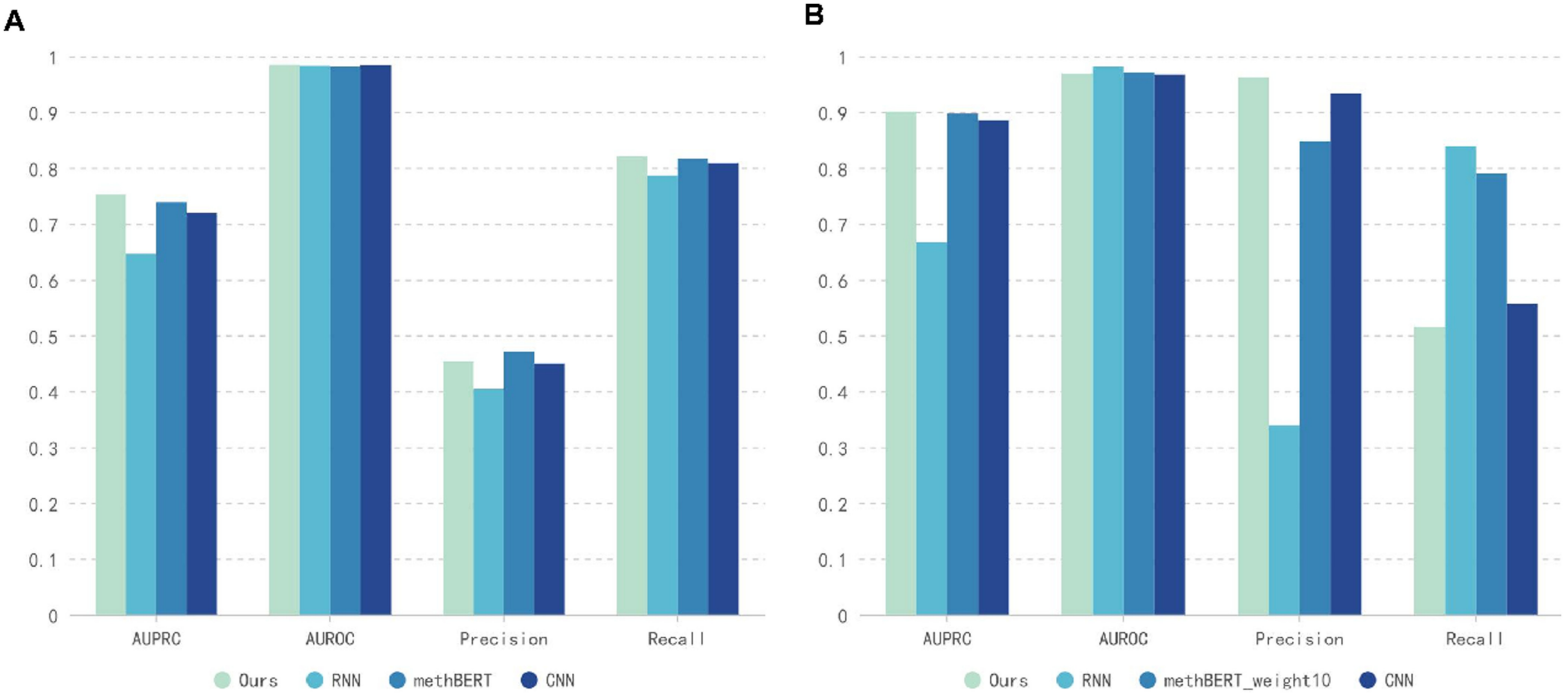
Comparison of the performance of the NanoCon model and other models in the cross-species settings. (A) The results obtained by training on the *O.sativa* dataset and testing on the *A.thaliana* dataset. (B) The results obtained by training on the *A.thaliana* dataset and testing on the *O.sativa* dataset.

As illustrated in Fig. 4, our model is at the forefront along with methBERT in terms of AUPRC in the interspecies experiments, which may be attributed to the transformer architecture that assists the model in capturing more generalized features. The performance of the CNN model is remarkably similar to ours in both experiments, with the gap in all metrics not exceeding 2%, indicating a certain advantage over the RNN model. In Fig. 4A, the performance difference between our model and the methBERT model is minimal, yet in Fig. 4B, a significant disparity can be observed in the precision and recall metrics. When trained on the *A.thaliana* dataset and tested on the *O.sativa* dataset, our model and the CNN model exhibit higher precision and lower recall compared to the methBERT and RNN models. This suggests that our model prioritizes the correct identification of positive cases over detecting all positive cases. To some extent, these results demonstrate the robustness and stability of our model in distinguishing between methylated and unmethylated sites compared to other models.

**Figure 5.**
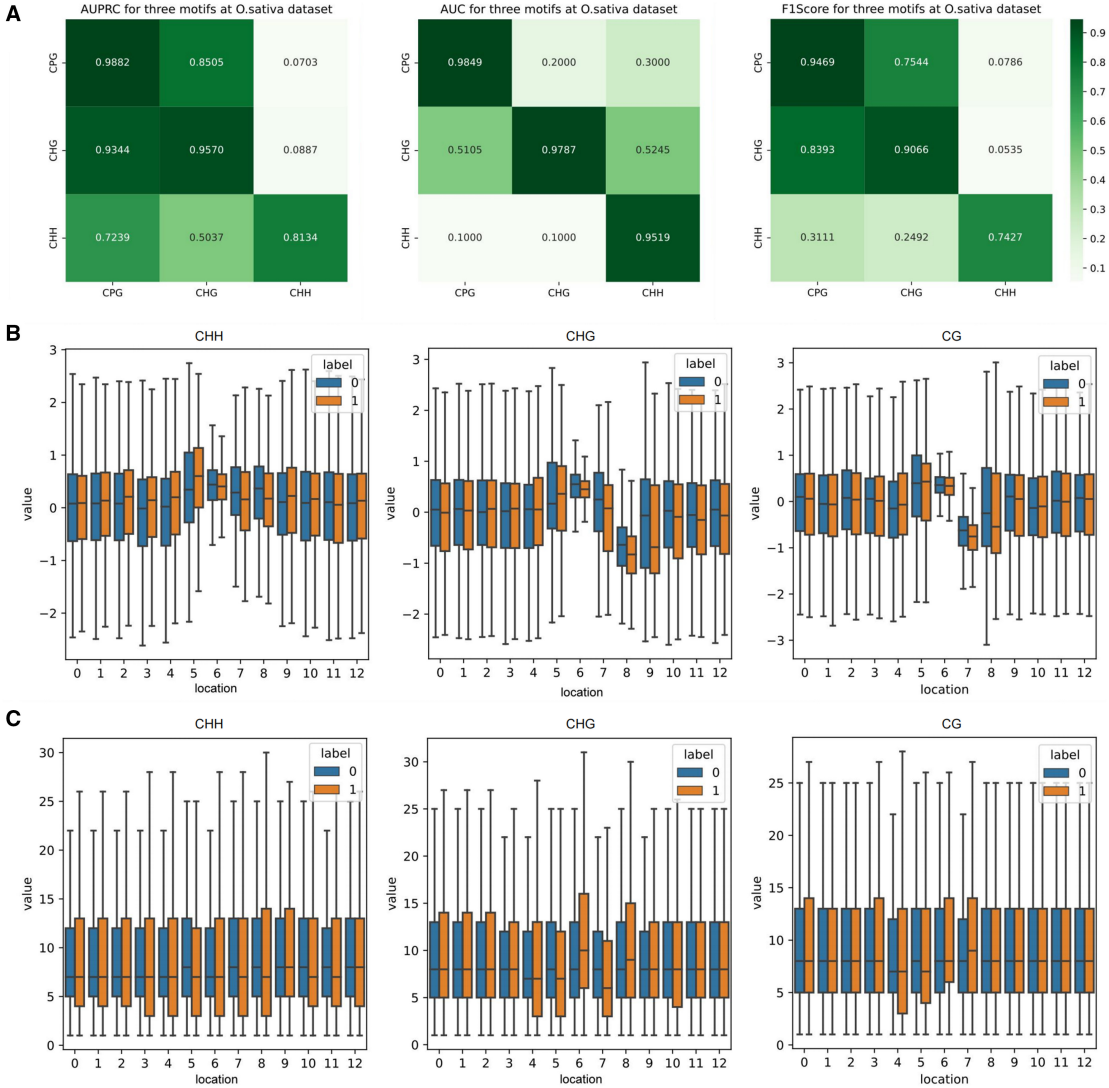
The results of cross-methylation motifs and the distributions for the mean value and length of electrical signals in the test set. (A) Heatmaps depicting the matrix composed of AUPRC, AUROC, and F1Score, obtained from training and testing on different motifs. Each unit represents the performance when using the motif from the corresponding row as the training set and the motif from the corresponding column as the testing set. (B and C) The distribution of mean and length of electrical signals in the test set corresponding to different motifs, presented using box plots.

In Fig. 5, we showcase the model's performance across different DNA methylation motifs, revealing varying prediction results. Figure 5A indicates the highest accuracy for CpG motifs, followed by CHG, with CHH motifs showing the lowest performance. Training on CpG and CHG motifs and testing on the other yields satisfactory results, but using CHH as the test set leads to suboptimal performance. This discrepancy may stem from the inherent differences in DNA sequences across motifs, impacting the model's effectiveness. In addition, electrical signal variances across motifs, depicted in Fig. 5B, suggest a more focused distribution for C and G in CHG and CpG, contrasting with the more varied CHH motif. This is further illustrated in Fig. 5C, where the CHH motif displays a broader distribution of positive samples compared to negative ones, unlike the other motifs.

### 3.4 Interpretability of models

To verify the representation learning ability and provide the interpretability of our model, we utilize the t-Distributed Stochastic Neighbor Embedding (t-SNE), a nonlinear dimensionality reduction algorithm that adeptly preserves the local structure of data for a visualized analysis of our model (Van Der Maaten 2014). By mapping high-dimensional data to a lower-dimensional space, the t-SNE chart reveals the distribution of data in this reduced dimensionality and we can discern how samples from different categories or clusters are distributed. In specific, for NanoCon model we visualized the output of the model's feature fusion module by t-SNE, in hopes of achieving an intuitive explanation of the model's predictive outcomes. Simultaneously, we obtained the feature matrices of the RNN, methBERT and CNN models.

**Figure 6.**
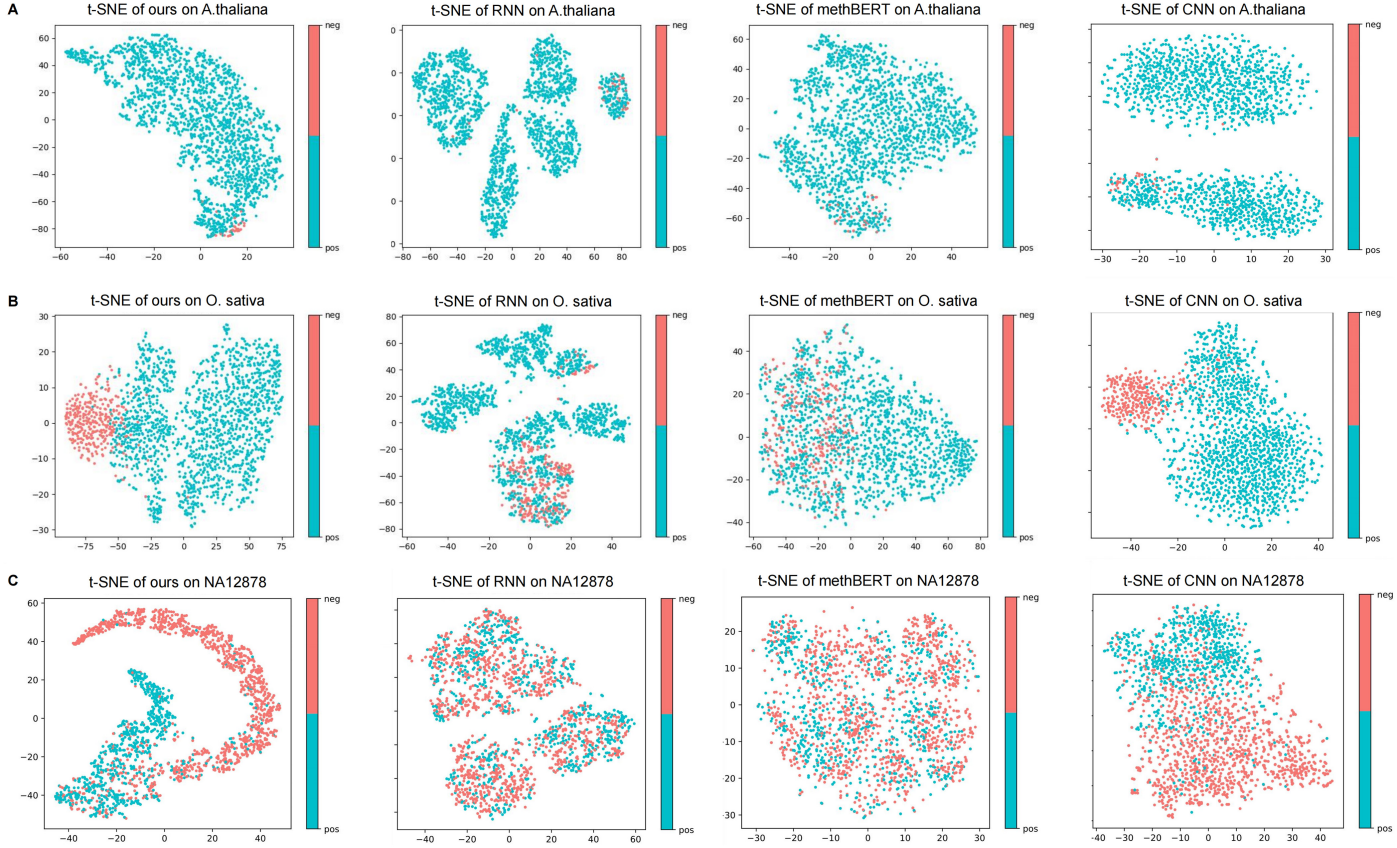
t-SNE visualization results. (A–C) The t-SNE visualization results for two datasets (*A.thaliana*, *O.sativa*, and *NA12878*) using four models (ours, RNN, methBERT, CNN). Each data point represents a sample, and the color indicates the category to which the sample belongs. The visualization aids in understanding the clustering behavior and the discriminative capability of the four models across different categories in these datasets.

In this section, we selected 2000 random samples from three distinct species datasets for testing. Leveraging our adeptly trained models, we acquired and then visualized feature representations using the t-SNE technique, as illustrated in Fig. 6A–C. Our method outperformed others in clustering, especially in differentiating positive and negative samples. This enhanced performance is likely attributable to the integration of a contrastive learning module in our framework, which proficiently captures inherent dataset patterns, thereby augmenting feature extraction and classification efficacy. Notably, on the *O.sativa* and *NA12878* datasets, CNN model showed better discriminability than RNN and methBERT models, as discussed earlier. This visualization analysis evaluates model performance in an interpretable manner, highlighting the strengths of our model in feature extraction and sample classification within imbalanced datasets.

## 4 Discussion and conclusion

In this study, we introduce NanoCon, a deep hybrid network leveraging contrastive learning, specifically tailored for methylation site detection in nanopore sequencing data. NanoCon integrates a transformer model for DNA sequence encoding with a Bi-GRU for electrical signal feature integration. We assessed its efficacy on datasets from *A.thaliana*, *O.sativa*, and humans, comparing it against other methods and conducting both ablation studies and cross-species, cross-motif performance evaluations. Our findings indicate that NanoCon excels in scenarios of significant data imbalance, demonstrating enhanced methylation site detection capabilities in these specific contexts. Moreover, our work sheds light on transferability and feature interplay, and underscores the potential of combining ensemble and deep learning approaches for further advancements in nanopore sequencing and bioinformatics.
